# Renal oncocytoma with liver metastasis: a case report with genetic analysis and literature review

**DOI:** 10.3389/fmed.2025.1558224

**Published:** 2025-04-30

**Authors:** Issa Al-kharouf, Carmen Miller, Ameer Hamza, Benyi Li, Da Zhang

**Affiliations:** ^1^Department of Pathology, University of Kansas Medical Center, Kansas City, KS, United States; ^2^Department of Urology, University of Kansas Medical Center, Kansas City, KS, United States

**Keywords:** oncocytoma, genetic profile, RGPD, NUP210L, PLCL1

## Abstract

Oncocytomas are clinically benign tumors composed of cells with abundant granular eosinophilic cytoplasm due to a high mitochondrial content. While typically non-metastatic, rare cases of metastatic oncocytomas have been documented. This report describes a unique case involving transcriptome analysis to identify genes associated with the oncocytoma signature. A 57-year-old woman presented to the emergency department with COVID-19 pneumonia. Incidentally, a CT chest scan revealed a large mass in the left upper quadrant. Further imaging of the abdomen and pelvis identified a 14 cm left renal mass and multiple low-density hepatic lesions. A liver biopsy confirmed a PAX-8 and CD10 positive carcinoma, consistent with metastatic renal cell carcinoma. Following neoadjuvant therapy, the patient underwent a left radical nephrectomy, partial hepatectomy, and cholecystectomy. This case emphasizes the rarity of metastatic oncocytoma and the importance of genomic testing in elucidating its molecular underpinnings. By identifying specific genetic alterations linked to the oncocytoma signature, genomic analysis offers critical insights into potential mechanisms of metastasis. These findings could enhance diagnostic accuracy and guide the development of targeted therapeutic strategies in rare metastatic cases of oncocytoma.

## Introduction

In the current 5th edition of the WHO Classification of Urinary and Male Genital Tumors, renal oncocytoma (RO) is classified as a subtype of oncocytic and chromophobe renal tumors. It is a benign neoplasm and typically asymptomatic (1, 2). Microscopically, RO displays nested or tubulocystic structures lined with eosinophilic cells containing granular cytoplasm. Although RO cells exhibit benign histological features, the presence of mitotic figures is still consistent with an oncocytoma diagnosis (3, 4). The nuclei are round with uniform chromatin and a single prominent nucleolus, though occasional areas of cellular atypia with large, irregularly contoured nuclei and smudged chromatin may be observed (5).

RO diagnosis is generally based on morphology, but distinguishing it from the eosinophilic variant of chromophobe renal cell carcinoma (eo-ChRCC) can be difficult. In these cases, immunohistochemistry aids in differentiation. CK7 is commonly employed, with focal staining in < 5% of cells supporting a diagnosis of RO (4). Additional markers such as kidney-specific cadherin and S100A1 may also be used, showing cytoplasmic and nuclear/cytoplasmic staining patterns, respectively, in RO (5, 6). For ambiguous cases or rare instances of metastatic RO, molecular analysis can provide further diagnostic clarity. Notable molecular signatures in RO include 11q13 rearrangements and losses of chromosomes 1, X, or Y (7). Genomic and transcriptomic analyses have demonstrated potential in distinguishing RO from chromophobe RCC (8, 9). The classification of oncocytic renal tumors remains a subject of ongoing refinement and debate. The Genitourinary Pathology Society (GUPS) recently proposed the term “oncocytic renal neoplasm of low malignant potential, not further classified” for solitary, sporadic tumors with equivocal RO/eo-ChRCC features but an indolent clinical course (10).

Although RO is generally benign, rare cases of metastasis have been reported in the literature (2, 11, 12). One notable biopsy-confirmed case reported by Perez-Ordonez et al. in 1997 described a patient with stable metastatic disease 58 months after diagnosis, managed expectantly (2). Additional reports by Oxley et al. (11) and others highlight similar occurrences, though some were published before chromophobe RCC was widely recognized, and not all were biopsy-confirmed. This raises uncertainty regarding whether reported metastases originated from the renal tumor or a separate primary oncocytoma. Molecular analysis of both primary and metastatic lesions presents a promising approach to clarify this distinction.

## Case presentation

A 57-year-old woman presented to the emergency department with COVID-19 pneumonia. Her past medical history included basal cell carcinoma. Incidentally, a chest CT revealed a large mass in the left upper quadrant. A follow-up CT of the abdomen and pelvis identified a 14.8 cm left renal mass and multiple low-density hepatic lesions. A liver biopsy confirmed PAX-8 positive carcinoma, consistent with metastatic renal cell carcinoma.

The patient began first-line systemic chemotherapy with combination Nivolumab and Ipilimumab, followed by Nivolumab monotherapy, aiming for tumor debulking and subsequent surgical resection. Despite stable hepatic lesions, a lack of significant therapeutic response led to second-line chemotherapy with Axitinib. After 8 weeks, follow-up imaging showed a slight reduction in the renal mass and stable hepatic lesions. However, subsequent scans indicated poor overall response, with mild progression of liver metastases.

**Figure 1:**
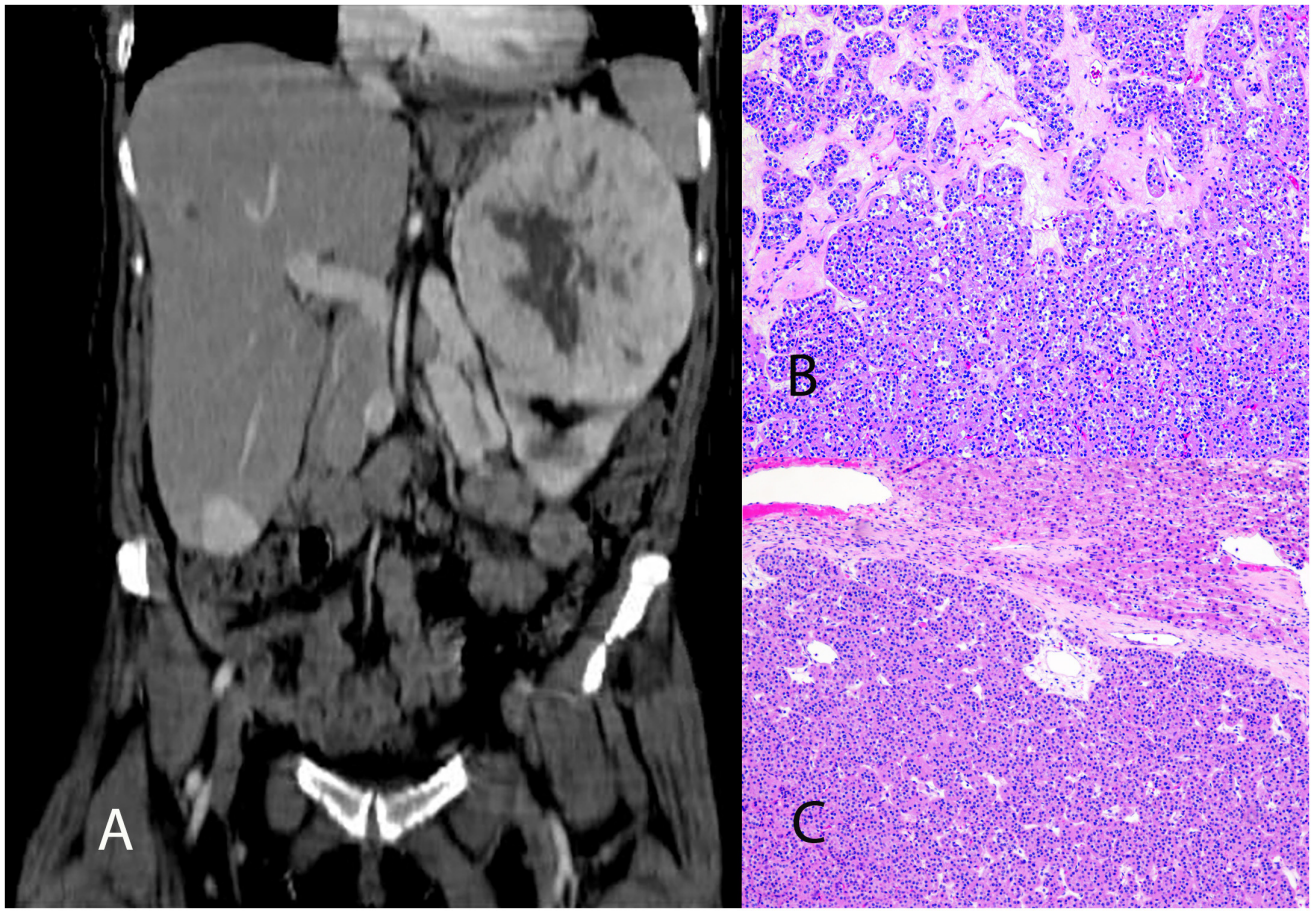
**(A)** Abdominal CT image showing a large, enhancing left upper pole renal mass measuring 14.8 cm and an enhancing liver lesion measuring 3 cm at its greatest dimension. **(B)** Histologic section of the renal mass displaying solid nests of oncocytic cells with granular, eosinophilic cytoplasm, interspersed with areas of hypocellular, myxoid to fibrous edematous stroma. **(C)** Histologic section of the liver mass revealing similar morphology and adjacent normal liver tissue.

**Figure 2:**
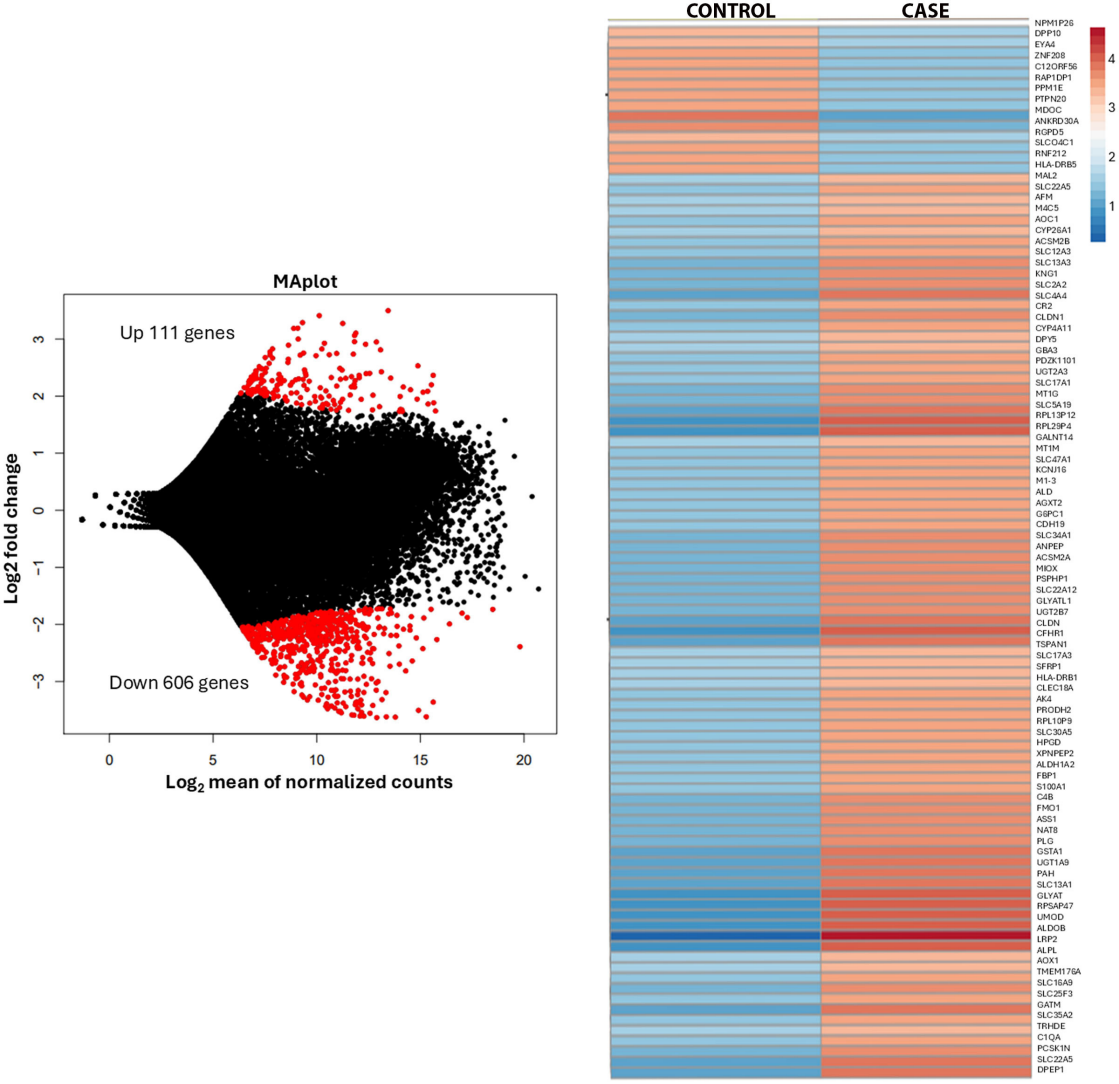
Transcriptome analysis of the renal mass.

The patient underwent left radical nephrectomy, partial hepatectomy, and cholecystectomy, with good surgical tolerance despite post-operative left lower lobe pneumonia. Histological analysis confirmed metastatic oncocytoma, with no evidence of lymphovascular invasion. The tumor showed solid nests of oncocytic cells with granular eosinophilic cytoplasm and areas of hypocellular myxoid-to-fibrous stroma (Figure 1). Immunohistochemistry showed positivity for PAX-8, CD117, patchy AMACR, and rare CK7-positive cells Intact SDHB and FH expression was noted. Electron microscopy on paraffin-embedded tissue revealed numerous poorly preserved intracellular organelles resembling mitochondria (Supplementary Figure 1). Transcriptome analysis of the renal mass identified 27,715 genes (performed at Admera Health, LLC, South Plainfield, NJ, USA). Compared to oncocytoma control specimens with no history of metastasis, the renal mass demonstrated 182 upregulated genes enriched in 32 pathways, with key genes of interest including RGPD6, RGPD5, RGPD8, RGPD2, NUP210L, and PLCL1. Additionally, 606 genes were downregulated, enriched in 20 pathways (Figure 2).

Three months post-operation, imaging revealed two new liver metastases, later determined to have likely been present for 5 months and slow-growing. Biopsy of these lesions showed no evidence of neoplasm. The patient underwent microwave ablation, though one hepatic lesion persisted, requiring two rounds of bland embolization. After 4 months, imaging showed slight growth of the lesion, and repeat microwave ablation is now planned.

## Discussion

This case highlights a rare instance of metastatic oncocytoma, challenging the conventional understanding of these typically benign tumors. The presence of metastatic liver lesions in a patient with renal oncocytoma underscores the importance of further research into the molecular mechanisms that may drive metastasis in oncocytomas.

The rarity of metastatic oncocytomas presents significant clinical management challenges, as no standardized treatment guidelines currently exist, and therapeutic options remain limited. In this case, the patient underwent first-line treatment with a combination of Nivolumab and Ipilimumab, followed by second-line Axitinib, along with surgical intervention. Incomplete tumor resection necessitated additional procedures, including embolization and microwave ablation.

Transcriptome analysis revealed distinct gene expression alterations compared to control oncocytoma samples. Notably, multiple genes encoding structural extracellular matrix glycoproteins, collagens, and proteoglycans were upregulated, including RGPD6, RGPD5, RGPD8, RGPD2, NUP210L, and PLCL1. The RGPD gene family, encoding RanBP2-like and GRIP domain-containing proteins, is emerging as a key regulator of extracellular matrix (ECM) remodeling, nuclear-cytoplasmic transport, and mechanotransduction, all of which are crucial for tumor metastasis. Alongside RGPD genes, NUP210—another nuclear pore complex component—plays a critical role in facilitating metastatic potential by maintaining nuclear pore integrity, modulating chromatin architecture, and enabling tumor cells to respond to mechanical stress (13). Upregulation of RGPD6, RGPD5, RGPD8, RGPD2, NUP210L, and PLCL1 has been observed in invasive tumors, implicating this functional network in the remodeling of ECM stiffness and integrin signaling, both of which enhance cancer cell migration (14). Nuclear pore dysregulation, including that mediated by RGPD proteins, alters the export and import of transcriptional regulators involved in epithelial-mesenchymal transition (EMT), enabling cells to transition toward a more mesenchymal, invasive phenotype (15). In addition, altered nuclear mechanics mediated by NUP210 and RGPD family members contributes to invadopodia formation, a key mechanism driving metastatic spread (13). Together, these findings position the RGPD-NUP210 axis as a critical driver of metastatic competence, highlighting it as a potential therapeutic target in metastatic cancer. Immunohistochemical staining did not show marked expression differences compared to the surrounding normal renal parenchyma.

In contrast, the downregulated pathways were associated with retinol, lipid, fatty acid, and amino acid metabolism, as well as xenobiotic metabolism via cytochrome P450 and proximal tubule transport. These findings suggest a distinct metabolic profile in metastatic oncocytomas, potentially distinguishing them from typical benign cases. Further analysis of these genes is currently in progress.

A literature review revealed 22 documented cases of metastatic oncocytoma (Supplementary Table 1) (20), six of which were surgically confirmed using various diagnostic methods, including immunohistochemistry, electron microscopy, FISH, SNP-based karyotyping, multiregion sequencing, and expression array analysis. Notably, in five of these six cases (83.3%), liver involvement was observed. While there is limited data on hepatic metastasis in renal oncocytomas, uveal melanoma serves as a prototypical neoplasm with a strong predilection for liver metastases, occurring in up to 95% of systemic cases (16). Studies by Ye et al. (17), Economou et al. (18), and Li et al. (19) have demonstrated that pathways involving HGF, c-Met, PI3K/Akt, and CXCR4 contribute to liver tropism in uveal melanoma. However, in our case, there was no evidence of increased expression of HGF, CXCR4, or activation of the PI3K/Akt pathway.

Our findings underscore the critical role of genomic testing in guiding clinical management and the potential for developing targeted therapies. Identifying specific genetic alterations in metastatic oncocytomas could pave the way for personalized treatment strategies and improved outcomes.

In conclusion, this case provides valuable insights into the genetic landscape of metastatic oncocytomas and emphasizes the importance of comprehensive genomic analyses. Identifying genetic alterations linked to metastasis offers promising avenues for future research and may lead to the development of targeted therapies for this rare and difficult-to-treat condition.

## Data Availability

The datasets presented in this study can be found in online repositories. The names of the repository/repositories and accession number(s) can be found in the article/Supplementary material.

## Supplementary material

Supplementary Figure 1 Electron microscopy performed on paraffin-embedded tissue revealed numerous poorly preserved intracellular organelles resembling mitochondria.

Supplementary Table 1 Literature review of previously reported metastatic renal oncocytoma cases.
