# Supplementary figures and images for: Renal oncocytoma with liver metastasis: a case report with genetic analysis and literature review
Source: Front Med (Lausanne). 2025 Apr 30;12:1558224. doi: 10.3389/fmed.2025.1558224 (PMC12075548; doi:10.3389/fmed.2025.1558224)

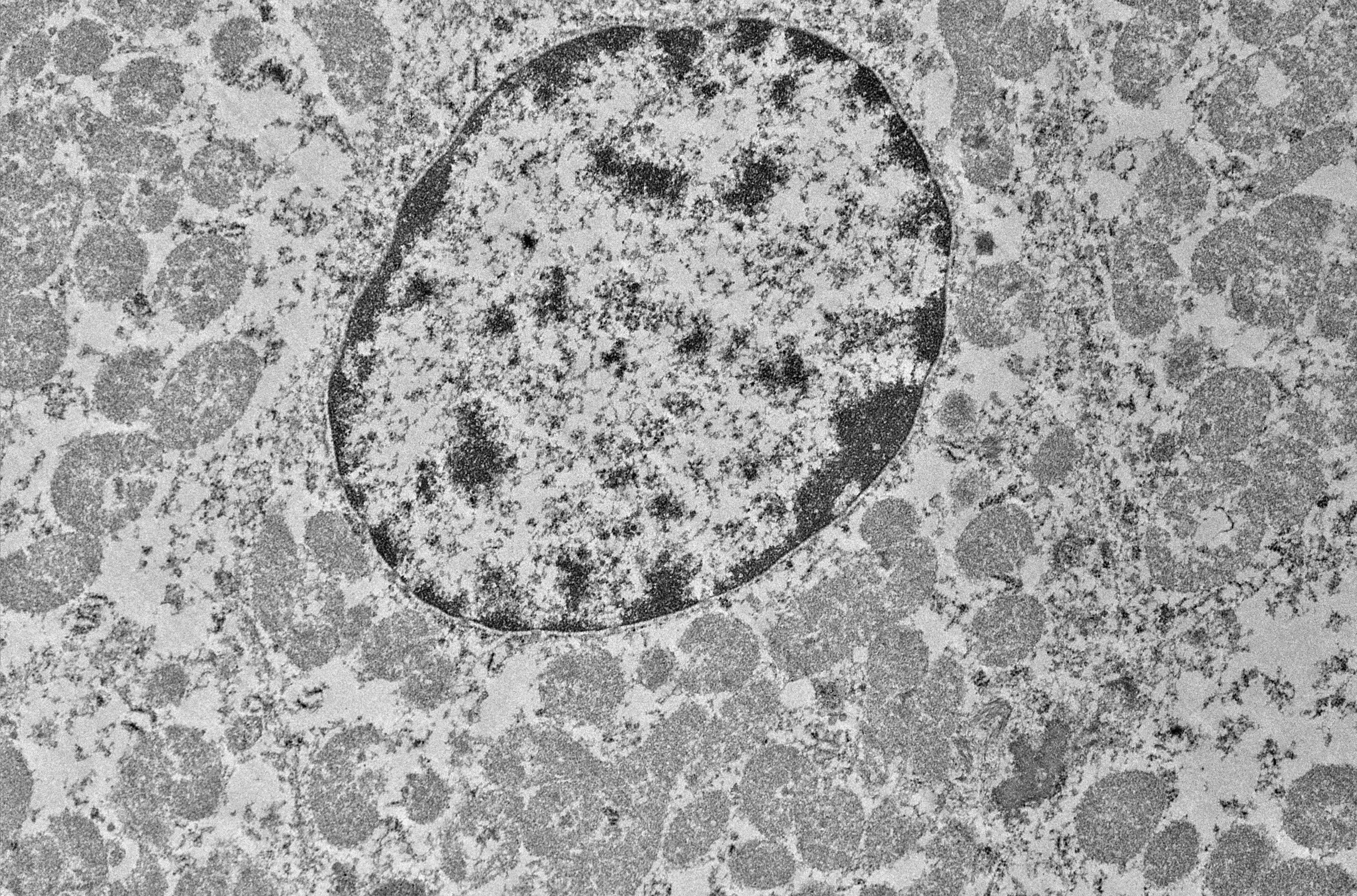

Supplement: Supplementary Figure 1 — Electron microscopy performed on paraffin-embedded tissue revealed numerous poorly preserved intracellular organelles resembling mitochondria. [file Image_1.jpeg]
